# Gas-Forming Pyogenic Liver Abscess with Septic Shock

**DOI:** 10.1155/2015/632873

**Published:** 2015-05-24

**Authors:** Muhammad S. Khan, Muhammad K. Ishaq, Kellie R. Jones

**Affiliations:** ^1^Department of Medicine, University of Oklahoma Health Sciences Center, Williams Pavilion 1130, P.O. Box 26901, Oklahoma City, OK 73190, USA; ^2^Department of Pulmonary & Critical Care Medicine, University of Oklahoma Health Sciences Center, Williams Pavilion 1130, P.O. Box 26901, Oklahoma City, OK 73190, USA

## Abstract

The pyogenic liver abscess caused by *Clostridium perfringens* (*C. perfringens*) is a rare but rapidly fatal infection. The main virulence factor of this pathogen is its *α*-toxin (lecithinase), which decomposes the phospholipid in cell membranes leading to cell lysis. Once the bacteria are in blood stream, massive intravascular hemolysis occurs. This can present as anemia on admission with evidence of hemolysis as indicated by low serum haptoglobin, high serum lactate dehydrogenase (LDH), elevated indirect bilirubin, and spherocytosis. The clinical course of *C. perfringens* septicemia is marked by rapidly deteriorating course with a mortality rate ranging from 70 to 100%. The very rapid clinical course makes it difficult to diagnose on time, and most cases are diagnosed at autopsy. Therefore it is important to consider *C. perfringens* infection in any severely ill patient with fever and evidence of hemolysis. We present a case of seventy-seven-year-old male with septic shock secondary to pyogenic liver abscess with a brief review of existing literature on *C. perfringens*.

## 1. Introduction

Pyogenic liver abscess caused by* Clostridium perfringens* is a rare disease and progression to septicemia has been associated with adverse outcomes. Early recognition and intervention are cornerstone of management due to aggressive course of the disease. This paper discusses an interesting case of elderly gentleman presenting with septic shock secondary to gaseous pyogenic liver abscess and also provides a brief review of the current literature on the topic.

## 2. Case

**Figure 1:**
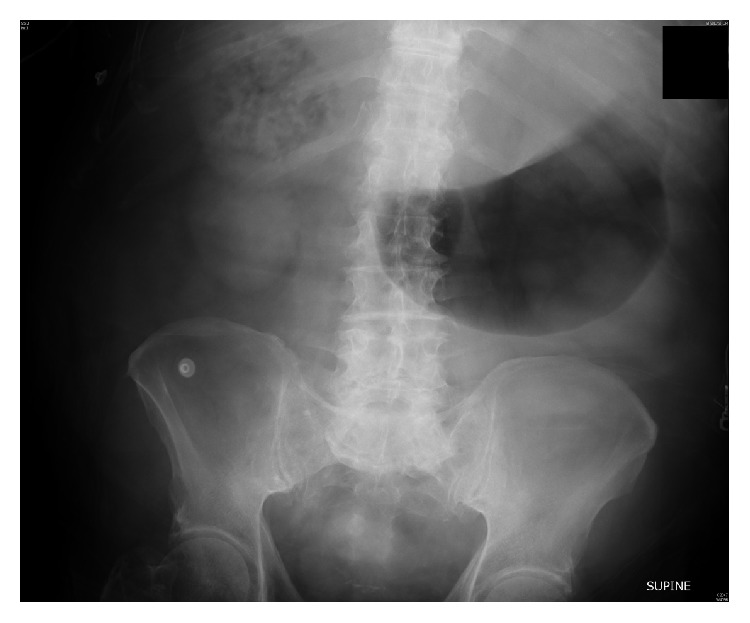
Abdominal radiograph showing right upper quadrant gas bubbles.

A seventy-seven-year-old native American male with past medical history of hypertension and diabetes mellitus presented at an outside hospital with two-day history of fever, generalized fatigue, nausea, vomiting, and abdominal pain. The patient was in usual state of health prior to presentation and was attending a church camp at a lake. He began to have nonbloody vomiting, nausea, and abdominal pain. He was checked by the camp nurse and was found to have a temperature of 101.7 degrees Fahrenheit. He was taken to the emergency room at a nearby hospital where he was found to be hypotensive with systolic blood pressure in 80s and subsequently transferred to the University of Oklahoma Medical Center for higher level of care. On arrival, the patient was confused. His temperature was 95 degrees Fahrenheit, blood pressure was 85/40 mmHg, pulse was 120/min, respiratory rate was 30/min, and oxygen saturation was 90% on room air. On examination, the patient was markedly confused and had cold and clammy extremities, pallor, and severe right upper quadrant pain on deep palpation coupled with abdominal distension. Significant labs on admission consisted of white blood cell count (WBC) of 33 K/cc^3^ (normal range 4–11 K/cc^3^), hemoglobin of 6 g/dL (normal range Hb 13–18 g/dL), platelet count of 278 k/cc^3^ (normal range 140–440 k/cc^3^), creatinine of 2.9 mg/dL (normal range 0.8–1.1 mf/dL), total bilirubin of 9.5 mg/dL (normal range 0.3–1.2 mg/dL), aspartate aminotransferase (AST) of 1179 IU/L (normal range 8–41 IU/L), alanine aminotransferase (ALT) of 370 IU/L (normal range 12–48 IU/L), alkaline phosphatase (AlkP) of 238 IU/L (normal range 70–178 IU/L), and lactic acid of 3.1 mmol/L (normal range 0.5–2.0 mmol/L). The patient was intubated on arrival due to inability to protect his airway. A central line was placed and normal saline (NS) fluid bolus was started. After three liters of NS was infused, the patient remained hypotensive and was started on norepinephrine infusion. Blood cultures, urine analysis, and urine cultures were obtained and the patient was initiated on broad spectrum antibiotics with intravenous (IV) vancomycin, piperacillin/tazobactam, and ciprofloxacin. Given marked abdominal tenderness and distension, an abdominal X-ray was obtained which showed radiographic findings concerning intra-abdominal abscess (Figure 1).

**Figure 2:**
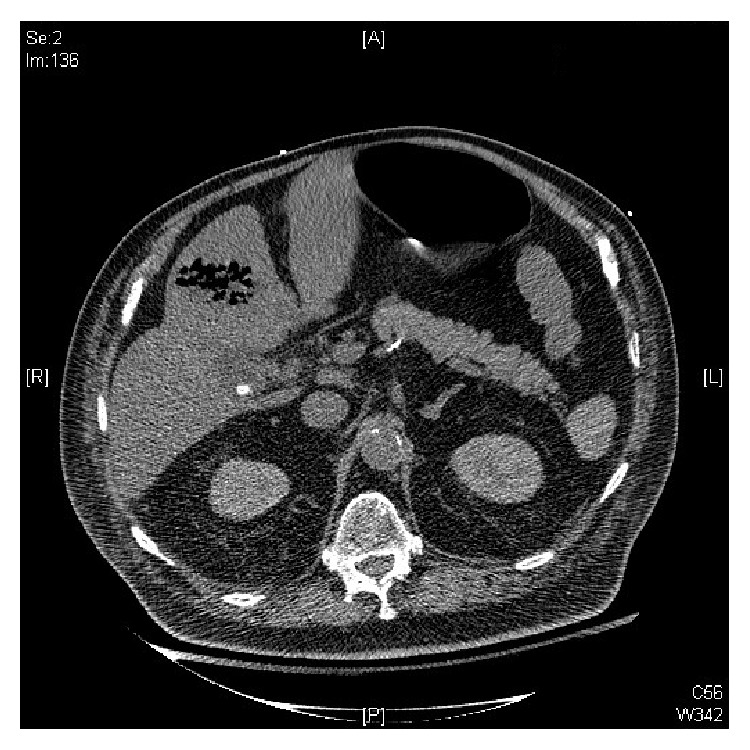
CT abdomen showing gas in left liver lobe.

Due to concern for liver abscess, a computed tomography scan (CT scan) of abdomen was done which revealed an ovoid air and debris containing structure in the left hepatic lobe concerning for hepatic abscess (Figure 2).

**Figure 3:**
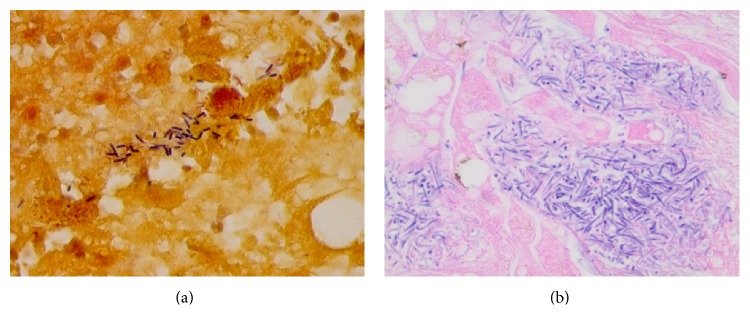
Pathology slides showing Gram positive rods in the liver parenchyma.

IV metronidazole was then initiated and surgery was consulted emergently. The patient required transfusion of packed red blood cells on arrival and his hemoglobin increased to 10 g/dL after transfusion but 3 hours later dropped to 6.4 g/dL. Lactate dehydrogenase (LDH) and haptoglobin were checked for concerns of hemolysis and were found to be 3442 U/L (normal range 112–236) and 90 mg/dL (normal range 38–195 mg/dL), respectively. A disseminated intravascular coagulation panel (DIC panel) was normal. Seven hours after admission the patient became bradycardiac with heart rate of 30 beats/min and quickly went into asystole. Cardiopulmonary resuscitation (CPR) was initiated and patient received multiple rounds of epinephrine during CPR. He developed ventricular tachycardia (VT) twice during CPR and was shocked without return of circulation. The resuscitation efforts were continued for 45 minutes and at that time the family indicated that they wanted no further resuscitative efforts. The family requested an autopsy limited to the abdominal cavity, which revealed a necrotic and hemorrhagic hepatic abscess with a myriad of Gram positive anaerobic rods in the liver parenchyma (Figure 3). The gallbladder had chronic cholecystitis and a black, friable calculus. The common bile duct was dilated without any evidence of ascending cholangitis. The blood cultures obtained on admission remained negative; therefore the cause of septic shock is unknown but we believe it was linked to gas-forming hepatic infection. The differential diagnosis of gas-forming anaerobic rods includes* Clostridium perfringens* and* Clostridium septicum*. Other common Gram negative gas-forming organisms include* Klebsiella pneumoiae* and* Escherichia coli*. However, due to anaerobic Gram negative rods on liver biopsy we believe that the most likely pathogen was* Clostridium* species.

## 3. Discussion

Gas-forming pyogenic liver abscess accounts for 7% to 24% of cases of pyogenic liver abscess cases [1]. The most common organism implicated is* Klebsiella pneumonia* which is a Gram negative rod and has a high fatality rate [1]. In patients with diabetes, the high levels of glucose may provide gas-forming microorganisms with a more favorable environment for forming gas via acid fermentation of glucose. In our case, even though the blood cultures remained negative, the morphology of organism on autopsy was most compatible with Gram positive anaerobic bacteria, raising the concern that this might represent* Clostridium* pyogenic liver abscess. Furthermore, the presence of gaseous pyogenic liver abscess and otherwise unexplained massive intravascular hemolysis with increased numbers of spherocytes and multiorgan failure supports the diagnosis of* Clostridium* septic shock. Our discussion in this paper will focus on* Clostridium perfringens* pyogenic liver abscess and septic shock.

*C. perfringens* is a Gram positive, anaerobic spore-forming bacterium which is responsible for a broad spectrum of human diseases resulting in considerable morbidity and mortality [2]. It is part of commensal flora of human digestive tract and female genital tract and is found in soil and fresh water sediments [3]. In cases of severe life threatening infections,* C. perfringens* can cause septicemia following food borne infection, wound associated soft tissue infection, and liver abscess, as well as lung abscess [4–7]. Risk factors include advanced age, diabetes mellitus, malignancy, liver cirrhosis, and immunocompromised states [8]. In some reports,* C. perfringens* infection has followed invasive procedure in the hepatobiliary and gastrointestinal tract, after central venous line insertion and routine gynecological procedures [9–11]. The septicemia may also occur without any underlying risk factors via bacterial translocation.

The mechanism of action is via secretions of toxins causing hemolysis and cell damage. Of these toxins, alpha-toxin is a major cause of hemolysis [12]. The alpha-toxin of* C. perfringens* has two domains with a loop in between. The N-terminal domain has phospholipase activity while C-terminal domain is hydrophobic in nature and enters into cell membrane [12]. The loop between C- and N-terminal contains GM1 ganglioside-binding motifs and specifically binds to GM1a. The phospholipase part disrupts the cell membrane phospholipids, while the alpha-toxin binding to GM1a triggers specific signaling events which leads to activation of tyrosine kinase A (TsKA) and the subsequent signaling cascade results in the release of tumor necrosis factor-alpha (TNF-A) which causes catastrophic hemolysis and inflammation [13]. Other virulence factors work primarily on vascular endothelium, causing capillary leakage. Like other clostridia,* C. perfringens* grows rapidly with the doubling time of about 7 minutes [14].

The incidence of pyogenic liver abscess and septicemia from* C. perfringens* is rare. Kasai et al. reported 119 cases of gas-forming abscess in diabetic patients of which only 8 cases were positive for* Clostridium* [15]. Another study from Japan reported that, among 5011 blood samples that were positive for any bacteria, only 41 were positive for* Clostridium*. Of the 41 samples, 16 were confirmed as septicemia, and 9 of the 16 were positive for* C. perfringens* [16]. The incidence of* C. perfringens* septicemia was reported to be 0.7 in 100000 per year in Canada [17]. Similarly, in a large hospital based study of 7989 positive blood cultures, only 7 cases of* C. perfringens* infection were reported [18].

The clinical course of* C. perfringens* septicemia and liver abscess is usually aggressive with high mortality ranging up to 70% to 100% [19]. The initial presentation can be variable, but fever and abdominal pain are common symptoms [16]. The abdominal pain has been strongly associated with a ruptured liver abscess. Once the bacteria are in blood stream, massive intravascular hemolysis occurs [20–22]. This can present as anemia on admission with evidence of hemolysis as indicated by low haptoglobin, high LDH, elevated indirect bilirubin, and spherocytosis [23]. Therefore, it is important to consider* Clostridium perfringens* infection in severely ill patient with fever and evidence of hemolysis.

Overall, the infection carries significant morbidity and mortality and early intervention has been shown to decrease mortality. Once the infection is suspected based on clinical picture, aggressive management with timely debridement of the focus, early initiation of antibiotics, and a multidisciplinary team approach is necessary [16]. Recently, van Bunderen et al. reported 40 cases of* C. perfringens* septicemia and hemolysis between 1990 and 2010 and the mortality rate was found to be 80% [24]. Law and Lee reported a review of 20 cases of which only 6 patients survived [23]. Among the patients who survived in both reports, an early attempt to remove the focus of infection was strongly associated with favorable outcomes [23, 24]. Similarly, Kurasawa et al. reported a review of 30 cases of* C. perfringens* liver abscess in which only 3 patients survived after receiving early surgical intervention via laparotomy and drainage [16]. The median time between admission and death varied between 6 and 11 hours signifying the aggressive course of disease [16, 23, 24].

There are no clear guidelines regarding any specific antibiotic use against* C. perfringens* infection. The antibiotics classified as appropriate for* Clostridium* are penicillin G, clindamycin, cefoxitin, metronidazole, ampicillin/sulbactam, piperacillin/tazobactam, and imipenem/cilastatin [25]. Patients who are treated with inappropriate antibiotics have significantly higher mortality rate of 75%, compared to patients treated with adequate antibiotic coverage [25]. Clindamycin, metronidazole, and rifampicin have been shown to be effective in reducing alpha-toxin release [26]. Additionally, the use of erythromycin pretreatment is thought to reduce TNF-alpha release from activated neutrophils, thus reducing hemolysis [27].

In summary,* C. perfringens* liver abscess and septicemia are a rare but lethal entity. Given the high mortality, index of suspicion should be very high in at-risk patients presenting with fever, unexplained hemolysis, and blood cultures positive for Gram positive rods. Early surgical intervention has been shown to be the most effective mode of treatment. In our patient the clinical course was very aggressive and, despite initiating appropriate antibiotics, the patient was never hemodynamically stable enough to permit surgery.
